# Supplementary material for: Itaconate transport across the plasma membrane and Salmonella-containing vacuole via MCT1/4 modulates macrophage antibacterial activity
Source: Nat Commun. 2025 Nov 26;16:10551. doi: 10.1038/s41467-025-65582-6 (PMC12658120; doi:10.1038/s41467-025-65582-6)
Supplement: Supplementary file 1 — Supplementary Information [file 41467_2025_65582_MOESM1_ESM.pdf]

Itaconate transport across the plasma membrane and *Salmonella*-containing vacuole via MCT1/4 modulates macrophage antibacterial activity

Qingcai Meng[1, *], Chengxi Li[1, *], Yuping Cai[2], Ying Chen[1], Xiaoqing Chen[1], Xin Wang[1], Biling Zhang[1], Yue Zhang[1], Feng Liu[1], Meixin Chen[1]✉

[1] Institute of Infectious Diseases, Shenzhen Bay Laboratory, Shenzhen, 518132, China.

[2] Interdisciplinary Research Center on Biology and Chemistry, Shanghai Institute of Organic Chemistry, Chinese Academy of Sciences, Shanghai, China.

✉Correspondence: chenmx@szbl.ac.cn

Table S1

Supplementary Figure 1-8

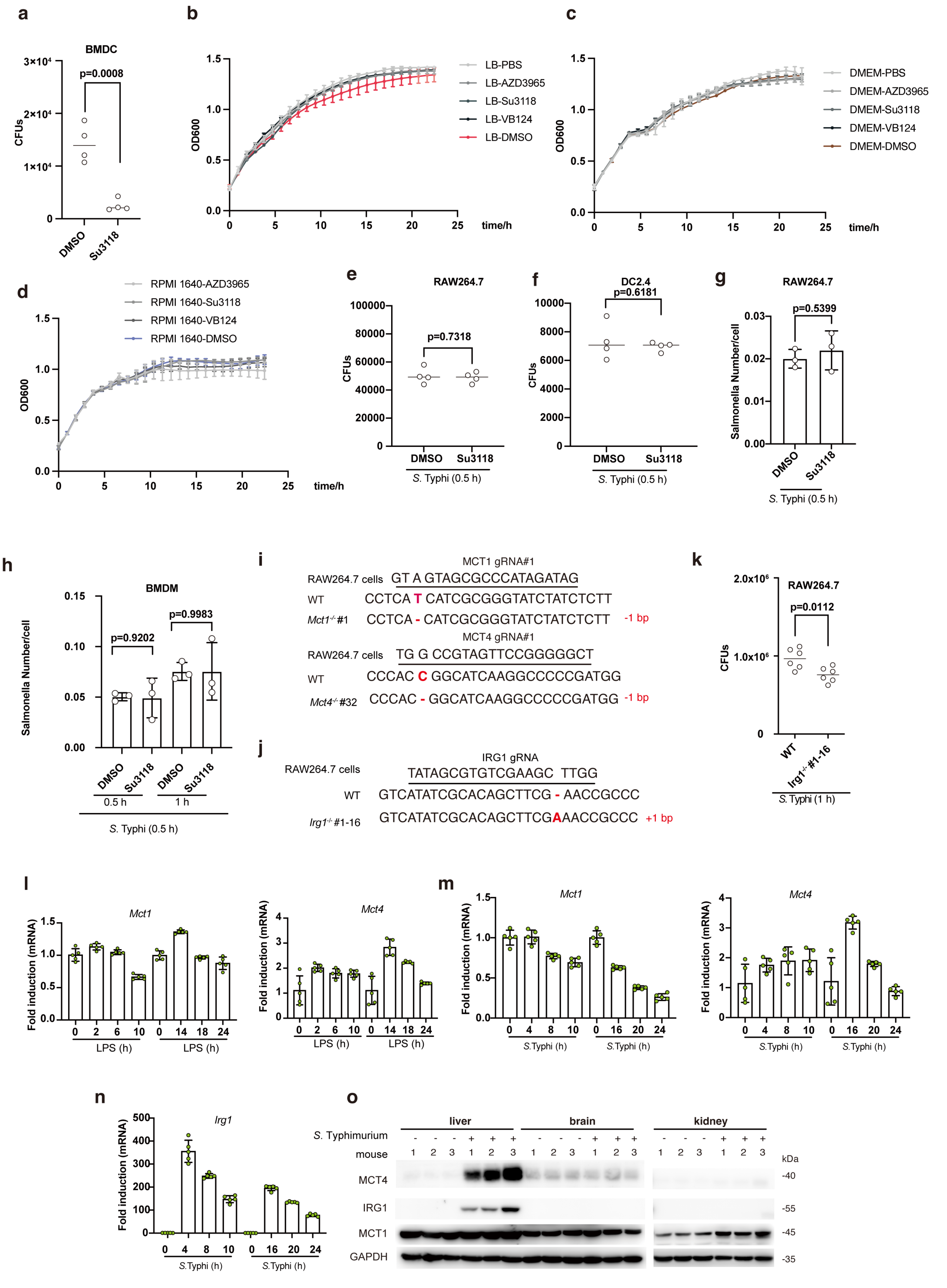
a
BMDC
p=0.0008
CFUs
DMSO
Su3118
b
OD600
time/h
LB-PBS
LB-AZD3965
LB-Su3118
LB-VB124
LB-DMSO
c
DMEM-PBS
DMEM-AZD3965
DMEM-Su3118
DMEM-VB124
DMEM-DMSO
d
RPMI 1640-AZD3965
RPMI 1640-Su3118
RPMI 1640-VB124
RPMI 1640-DMSO
e
RAW264.7
p=0.7318
S. Typhi (0.5 h)
f
DC2.4
p=0.6181
g
p=0.5399
Salmonella Number/cell
h
BMDM
p=0.9202
p=0.9983
0.5 h
1 h
i
MCT1 gRNA#1
RAW264.7 cells GT A GTAGCGCCCATAGATAG
WT CCTCA T CATCGCGGGTATCTATCTCTT
Mct1-/- #1 CCTCA - CATCGCGGGTATCTATCTCTT -1 bp
MCT4 gRNA#1
RAW264.7 cells TG G CCGTAGTTCCGGGGGCT
WT CCCAC C GGCATCAAGGCCCCCGATGG
Mct4-/- #32 CCCAC - GGCATCAAGGCCCCCGATGG -1 bp
j
IRG1 gRNA
RAW264.7 cells TATAGCGTGTCGAAGC TTGG
WT GTCATATCGCACAGCTTCG - AACCGCCC
Irg1-/- #1-16 GTCATATCGCACAGCTTCGAAACCGCCC +1 bp
k
p=0.0112
WT
Irg1-/- #1-16
S. Typhi (1 h)
l
Mct1
Mct4
Fold induction (mRNA)
LPS (h)
m
S.Typhi (h)
n
Irg1
o
liver
brain
kidney
S. Typhimurium
mouse
kDa
MCT4
IRG1
MCT1
GAPDH
-40
-55
-45
-35

Supplementary Fig. 1 The inhibition of MCT1 and MCT4 transport activity resists *Salmonella* infection. (a) BMDC (bone marrow-derived dendritic cells) treated with Su3118 (10 μM) were infected with *Salmonella* (*S.*) Typhi. The number of bacteria in cells was determined at 22 h after infection. **(b-d)** *S*. Typhimurium was grown in LB media (b), DMEM (c) or RPMI 1640 culture media (d), with Su3118 (10 μM), AZD3965 (1 μM) or VB124 (20 μM). The growth of *Salmonella* (OD600) was monitored for 24 h. **(e, f)** RAW264.7 cells (e) or DC2.4 cells (f) treated with Su3118 (0, 10 μM, 22 h) were infected with *S*. Typhi. The number of bacteria in cells was determined at 0.5 hour after infection. **(g, h)** RAW264.7 or BMDM pretreated with Su3118 (0, 10 μM, 22 h) were infected with *S*. Typhi stably expressing mScarlet. At the indicated time point, cells were fixed and stained with Hoechst. The number of intracellular *Salmonella* per cell was quantified with Opera Phenix Plus High Content Screening System. **(i)** Sequence alignment analysis of wild-type (WT), *Mct1*$^{-/-}$ or *Mct4*$^{-/-}$ RAW264.7 cells. **(j)** Sequence alignment analysis of WT, *Irg1*$^{-/-}$ RAW264.7 cells. **(k)** This is related to Figure 1k. WT or *Irg1*$^{-/-}$ RAW264.7 cells were infected with *S*. Typhi for 1 h. **(l-n)** Expression of MCT1, MCT4 in cells or mice after LPS treatment or bacterial infection. **(l, m)** The mRNA level of MCT1, MCT4 or IRG1 in RAW264.7 treated with LPS (l) or infected with *S*. Typhi (m, n) were measured by quantitative real-time PCR. **(o)** Immunoblot analysis of protein extracts from the indicated murine organs with MCT1, MCT4, IRG1 or GAPDH antibodies. Data are shown as the mean ± SD (g, h, l-n). Independent biological replicates-n=3 (b-c, g, h); n = 4 (a, e, f); n=5 (l-n); n=6 (k). A two-tailed Student's t-test was conducted for pairwise comparisons (a, e, f, i).

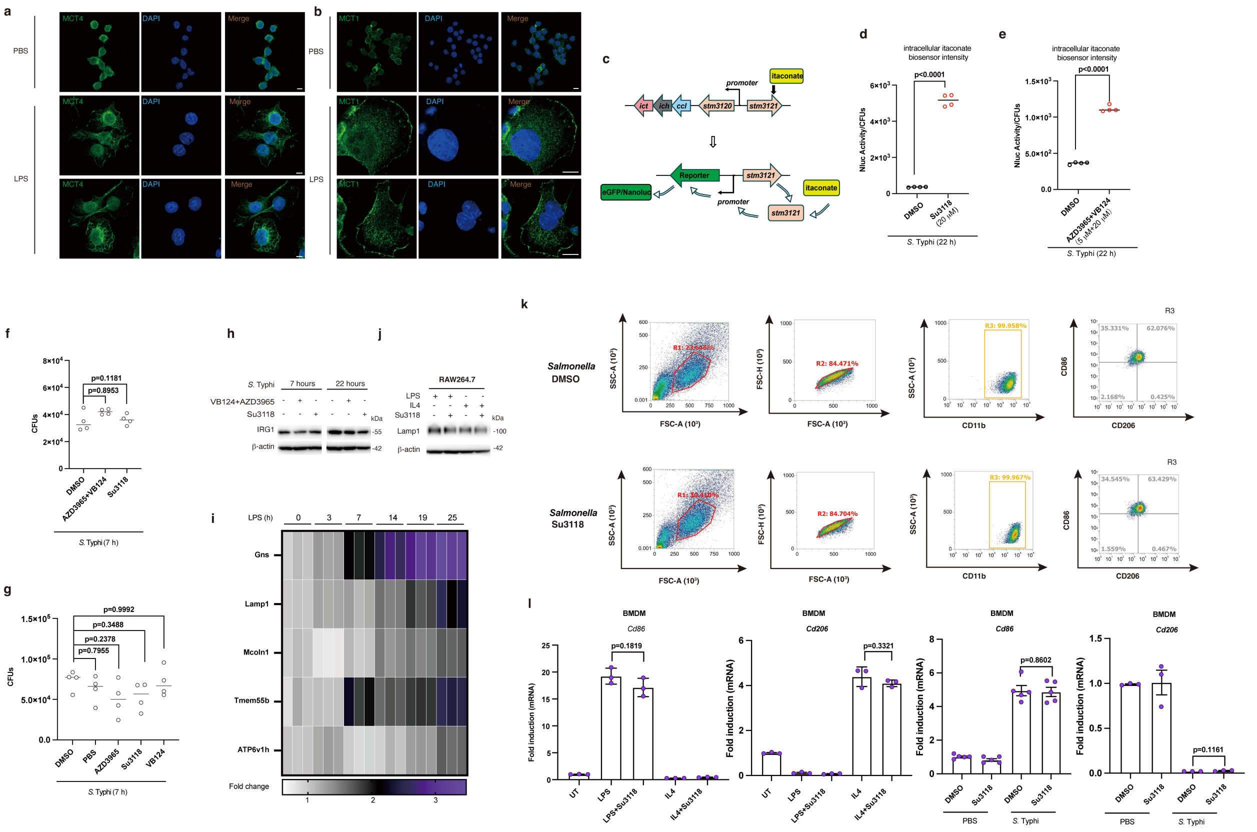

Supplementary Fig. 2 The inhibition of MCT1 and MCT4 transport activity promotes the prevalence of itaconate-targeted *Salmonella*. (a, b) The location of MCT1 and MCT4 in macrophages. RAW264.7 treated with LPS (100ng/ml) for 24 h were fixed, stained with DAPI (Blue) to visualize nuclei and incubated with MCT4 antibody (a) or MCT1 antibody (b) along with Alexa 488-conjugated anti-rabbit antibody (Green), and imaged under a confocal microscope. Scale bar, 10 μm. **(c)** The rationale of itaconate biosensor. **(d, e)** RAW264.7 cells treated with the indicated inhibitors (Su3118 (20 μM), AZD3965 (5 μM) + VB124 (20 μM)) were infected with *S*. Typhi (22 h) encoding the nanoluciferase-dependent itaconate biosensor. The levels of nanoluciferase in cell lysates were measured. **(f, g)** RAW264.7 treated with the indicated inhibitors were infected with *S.* Typhi. The number of bacteria in cells was determined at 7 h after infection. **(h)** Immunoblot analysis of protein extracts from RAW264.7 infected with *S*. Typhi for the indicated time points by using IRG1 and β-actin antibodies. **(i)** Relative mRNA levels of lysosomal genes in BMDM treated with Su3118 (5 μM, 24h) with or without LPS (10 ng/ml, the indicated time points) were determined by quantitative real-time PCR. **(j)** RAW264.7 cells treated with LPS (10 ng/ml, 24 h) or IL-4 (20 ng/ml, 24 h). The cell lysates were analyzed by immunoblotting with anti-Lamp1 and anti-β-actin. **(k)** Representative flow plot from BMDM showing CD11b, CD86, CD206 expression in BMDM infected with *S*. Typhi (24 h) with or without Su3118 (5 μM, 22 h) treatment. **(l)** Relative mRNA levels of the indicated cytokines in BMDM treated with Su3118 (5 μM, 22

h) with LPS (10 ng/ml, 18 h)/IL-4 (20ng/ml, 18 h) were determined by quantitative real-time PCR. Data are shown as the mean ± SD (l). Independent biological replicates-n = 3 (i, l); n=4 (d-g). A two-tailed Student's t-test was conducted for pairwise comparisons (d, e, l), while one-way ANOVA was used for multiple comparisons involving a single independent variable (f, g).

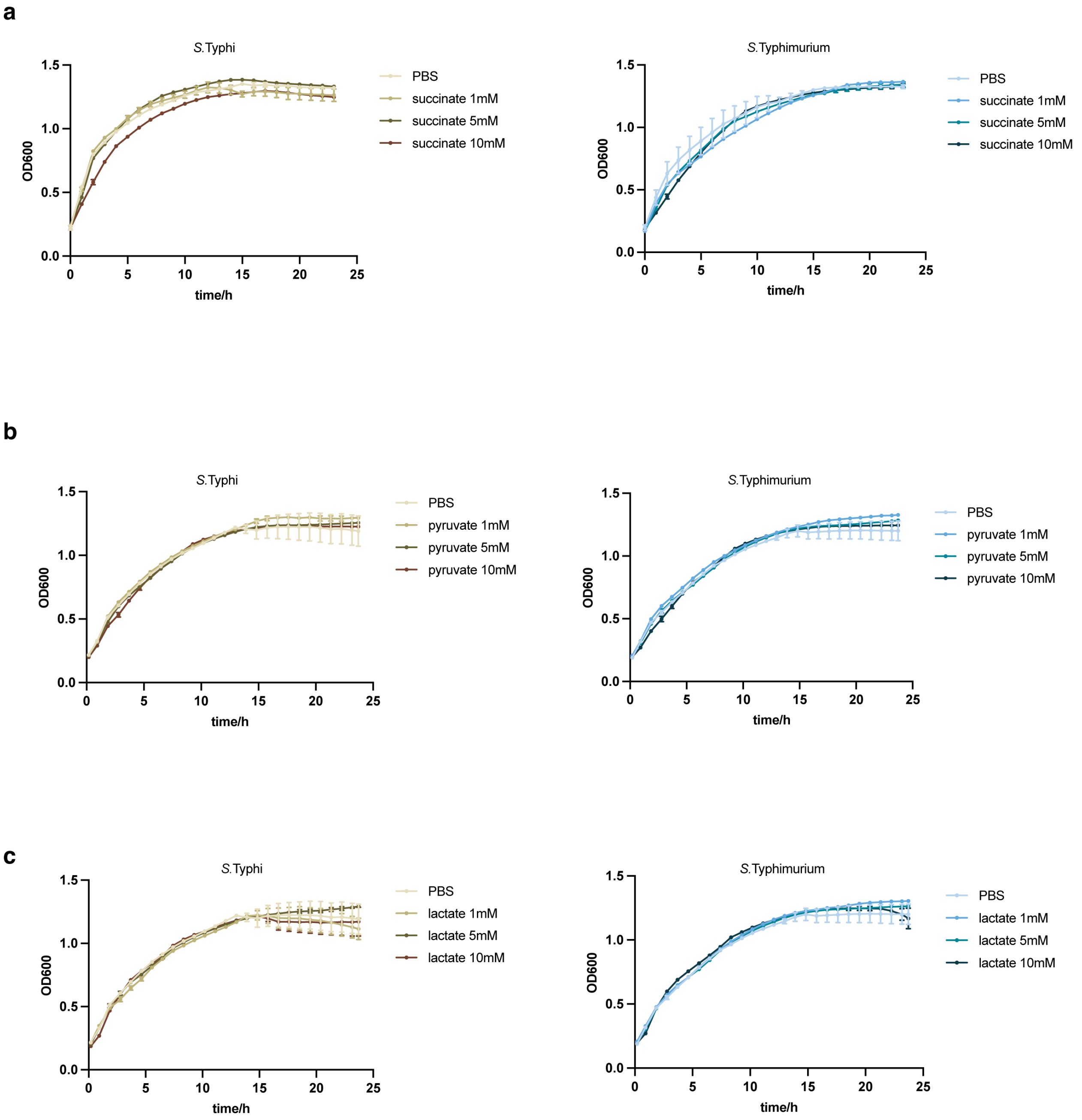

Supplementary Fig. 3 Succinate, pyruvate and lactate do not impair *Salmonella* growth. (a-c) *S*. Typhi or *S*. Typhimurium was incubated in LB containing different metabolites (succinate, pyruvate or lactate) with the indicated concentration. The growth of *Salmonella* (OD600) was monitored for 24 h in a microplate reader. Data in a-c is mean ± SD from 3 independent biological replicates.

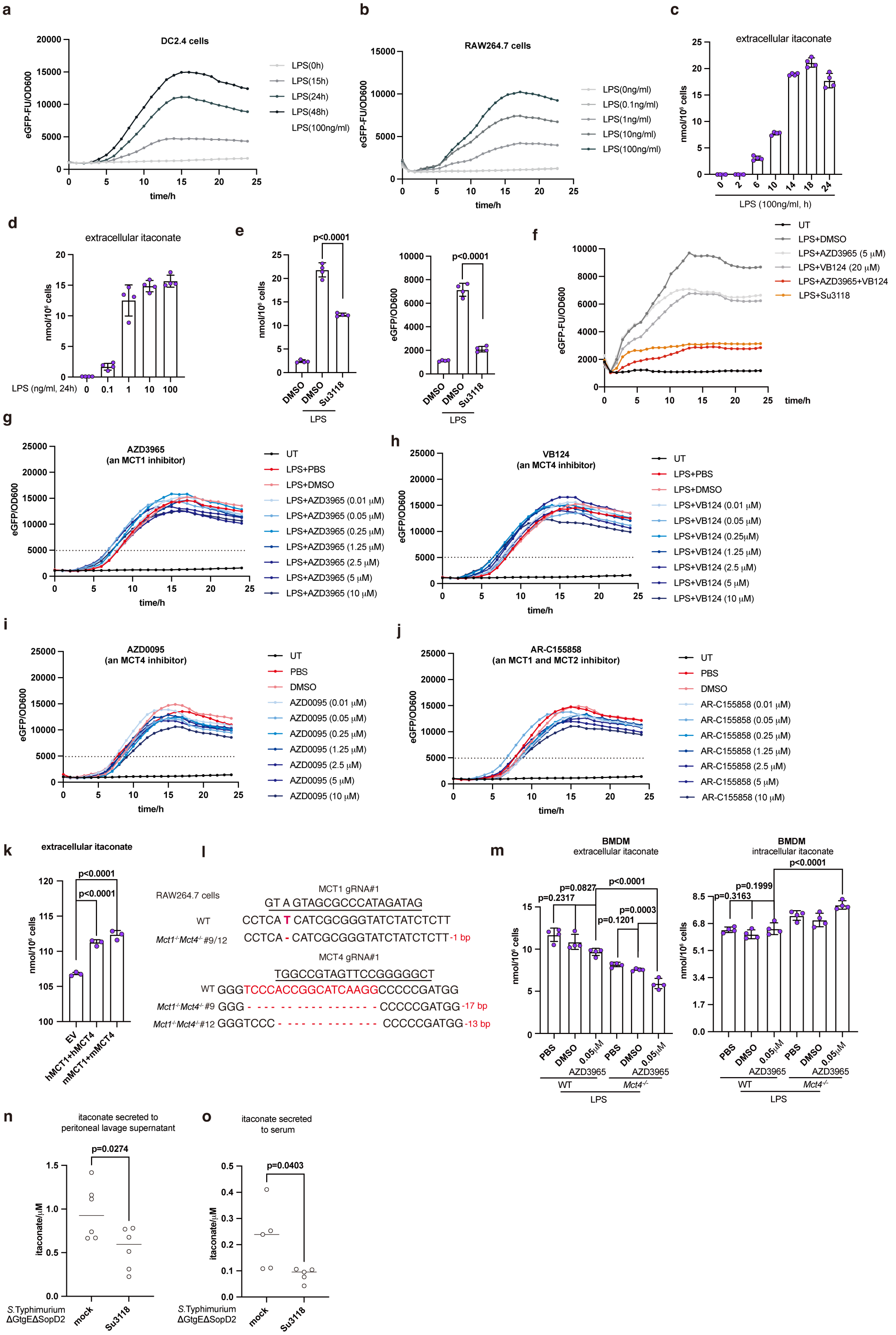

a
DC2.4 cells
eGFP-FU/OD600
time/h
LPS(0h)
LPS(15h)
LPS(24h)
LPS(48h)
LPS(100ng/ml)
b
RAW264.7 cells
eGFP-FU/OD600
time/h
LPS(0ng/ml)
LPS(0.1ng/ml)
LPS(1ng/ml)
LPS(10ng/ml)
LPS(100ng/ml)
c
extracellular itaconate
nmol/10^6 cells
LPS (100ng/ml, h)
d
extracellular itaconate
nmol/10^6 cells
LPS (ng/ml, 24h) 0 0.1 1 10 100
e
p<0.0001
nmol/10^6 cells
eGFP/OD600
DMSO DMSO Su3118
LPS
f
UT
LPS+DMSO
LPS+AZD3965 (5 μM)
LPS+VB124 (20 μM)
LPS+AZD3965+VB124
LPS+Su3118
eGFP-FU/OD600
time/h
g
AZD3965 (an MCT1 inhibitor)
eGFP/OD600
time/h
UT
LPS+PBS
LPS+DMSO
LPS+AZD3965 (0.01 μM)
LPS+AZD3965 (0.05 μM)
LPS+AZD3965 (0.25 μM)
LPS+AZD3965 (1.25 μM)
LPS+AZD3965 (2.5 μM)
LPS+AZD3965 (5 μM)
LPS+AZD3965 (10 μM)
h
VB124 (an MCT4 inhibitor)
eGFP/OD600
time/h
UT
LPS+PBS
LPS+DMSO
LPS+VB124 (0.01 μM)
LPS+VB124 (0.05 μM)
LPS+VB124 (0.25μM)
LPS+VB124 (1.25 μM)
LPS+VB124 (2.5 μM)
LPS+VB124 (5 μM)
LPS+VB124 (10 μM)
i
AZD0095 (an MCT4 inhibitor)
eGFP/OD600
time/h
UT
PBS
DMSO
AZD0095 (0.01 μM)
AZD0095 (0.05 μM)
AZD0095 (0.25 μM)
AZD0095 (1.25 μM)
AZD0095 (2.5 μM)
AZD0095 (5 μM)
AZD0095 (10 μM)
j
AR-C155858 (an MCT1 and MCT2 inhibitor)
eGFP/OD600
time/h
UT
PBS
DMSO
AR-C155858 (0.01 μM)
AR-C155858 (0.05 μM)
AR-C155858 (0.25 μM)
AR-C155858 (1.25 μM)
AR-C155858 (2.5 μM)
AR-C155858 (5 μM)
AR-C155858 (10 μM)
k
extracellular itaconate
p<0.0001
p<0.0001
nmol/10^6 cells
EV
hMCT1+hMCT4
mMCT1+mMCT4
l
RAW264.7 cells
MCT1 gRNA#1
GT A GTAGCGCCCATAGATAG
WT CCTCA T CATCGCGGGTATCTATCTCTT
Mct1-/-Mct4-/- #9/12 CCTCA - CATCGCGGGTATCTATCTCTT -1 bp
MCT4 gRNA#1
TGGCCGTAGTTCCGGGGGCT
WT GGGTCCCACCGGCATCAAGGCCCCCGATGG
Mct1-/-Mct4-/- #9 GGG - - - - CCCCCGATGG -17 bp
Mct1-/-Mct4-/- #12 GGGTCCC - - - - CCCCCGATGG -13 bp
m
BMDM extracellular itaconate
p=0.2317
p=0.0827
p<0.0001
p=0.0003
p=0.1201
nmol/10^6 cells
BMDM intracellular itaconate
p<0.0001
p=0.1999
p=0.3163
PBS DMSO 0.05μM AZD3965
WT
Mct4-/-
LPS
n
itaconate secreted to peritoneal lavage supernatant
p=0.0274
itaconate/μM
S.Typhimurium ΔGtgEΔSopD2
mock
Su3118
o
itaconate secreted to serum
p=0.0403
itaconate/μM
S.Typhimurium ΔGtgEΔSopD2
mock
Su3118

Supplementary Fig. 4 Itaconate secretion is detected by itaconate biosensor or LC-MS. (a, b) DC2.4 cells (a) were stimulated with LPS (100ng/ml). RAW264.7 (b) were stimulated with LPS (24h). The cell supernatant and *Salmonella* carrying itaconate eGFP biosensor were cultured in a 96-well plate. The intensity of eGFP was examined. **(c, d)** Detection of extracellular itaconate of RAW264.7 treated with LPS for the indicated doses or time points by LC-MS. **(e)** Extracellular itaconate of RAW264.7 cells treated with LPS (100ng/ml) and Su3118 (10 μM) were collected and subjected to be analyzed by itaconate biosensor (right) or LC-MS (left). **(f-j)** RAW264.7 cells stimulated with LPS (100 ng/ml) were treated with the indicated inhibitors (AZD3965, VB124, AZD0095, AR-C155858: 0-10 μM as indicated, VB124 20 μM in fig. f) for 24 h. The cell supernatant and *S.* Typhi carrying itaconate eGFP biosensor were cultured in a 96-well plate. The intensity of eGFP was examined. **(k)** Extracellular itaconate from HEK 293T cells stably expressing IRG1 transfected with Flag-MCT1, Flag-MCT4 were measured by LC-MS (h, human; m, mouse). **(l)** Sequence alignment analysis of WT, *Mct1*$^{-/-}$ *Mct4*$^{-/-}$ (#9, #12) RAW264.7 cells. **(m)** Itaconate from BMDM treated with AZD3965 (0, 0.05 μM) and LPS (100 ng/ml) for 24 h were determined by LC-MS. PBS and DMSO as controls, same groups in Fig. 3k, l. **(n, o)** C57BL/6 (WT) or *Mct4*$^{-/-}$ mice were injected Su3118 (5 mg/kg) intraperitoneally. 3 h later, mice were infected intraperitoneally with *S*. Typhimurium ΔGtgEΔSopD2 (10^4 CFU) for 24 h. The levels of itaconate in serum (n=5) or peritoneal lavage supernatant (n=6) were measured by LC-MS. The itaconate level in the peritoneal lavage supernatant represents a relative concentration, determined from 5 ml of PBS used to rinse the mouse peritoneal cavity. Data are shown as the mean ± SD (c-e, k, m). Independent biological replicates-n=3 (a, b, f-k); n=4 (c-e, m); n=6 in mock group and n=5 in Su3118 group in fig. n; n=5 (o). A two-tailed Student's t-test was conducted for pairwise comparisons (e, n, o), while one-way ANOVA was used for multiple comparisons involving a single independent variable (k, m).

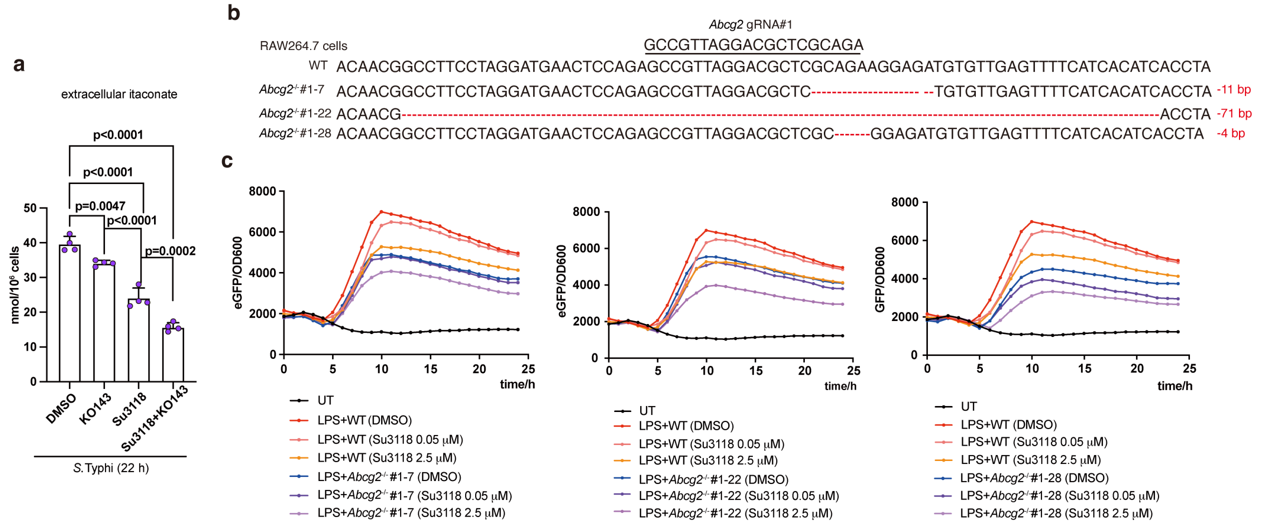

Supplementary Fig. 5 Inhibition of MCT1, MCT4 and ABCG2 abolishes the secretion of the majority of itaconate. (a) RAW264.7 cells infected with *S.* Typhi were treated with the indicated inhibitors (Su3118, 0/10 μM; KO143, 0/10 μM) for 22 h. The levels of secreted itaconate were measured by LC-MS. **(b)** Sequence alignment analysis of WT, *Abcg2*$^{-/-}$ (#1-7, #1-22, #1-28) RAW264.7 cells. **(c)** WT or *Abcg2*$^{-/-}$ RAW264.7 cells stimulated with LPS (100 ng/ml) were also treated with the indicated inhibitors (Su3118, 0, 0.05, 2.5 μM) for 24 h. The cell supernatant and *S.* Typhi carrying itaconate eGFP biosensor were mixed and cultured in a 96-well plate. The intensity of eGFP was examined by the plate reader. Data are shown as the mean ± SD (a). Independent biological replicates-n= 3 (c); n=4 (a). One-way ANOVA was used for multiple comparisons involving a single independent variable (a).

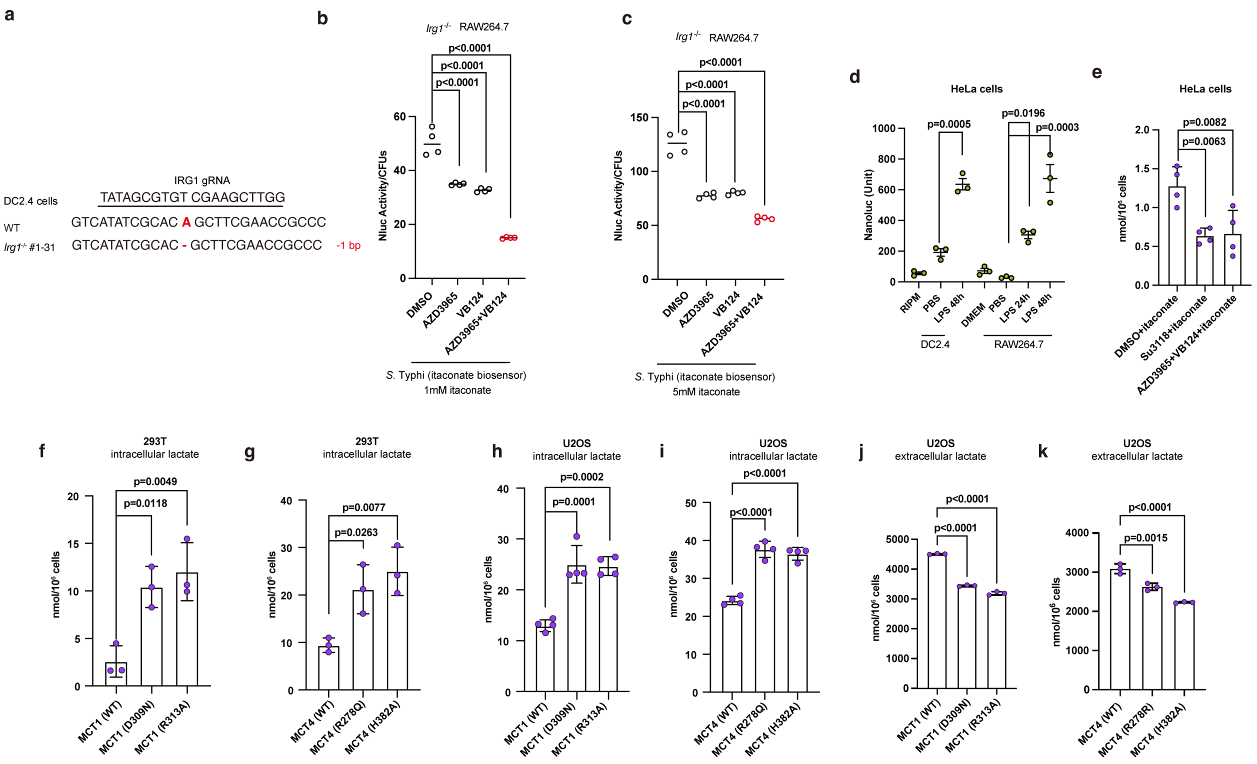

l

pH	monocarboxylic itaconate (%)
7.4	1.3%
7.2	2.1%
6.8	5.3%
6.5	10.1%
6.2	18.3%
6.0	26.2%
5.5	52.8%

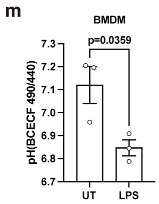

Supplementary Fig. 6 MCT1 and MCT4 transport itaconate into cells. (a) Sequence alignment analysis of WT and *Irg1*$^{-/-}$ DC2.4 cells. **(b, c)** The Entry of itaconate into cells is suppressed by the combinational VB124 (20 μM) and AZD3965 (1 μM). *Irg1*$^{-/-}$ RAW264.7 incubated with itaconate (1 mM or 5 mM) and treated with the indicated inhibitors were infected with *S.* Typhi carrying itaconate nanoluciferase biosensor for 22 h. The intensity of nanoluciferase activity was examined by the plate reader. **(d)** Itaconate released from LPS-treated DC2.4 or RAW264.7 cells entered HeLa cells. Cell supernatant from RAW264.7 or DC2.4 cells treated with LPS (100ng/ml) or PBS were collected and incubated with HeLa cells for 4 h. HeLa cells were washed with PBS for several times and infected by *S*. Typhi carrying itaconate nano-nanoluciferase biosensor for another 4 h. The levels of nanoluciferase in cell lysates were measured. RPMI 1640 or DMEM media are negative controls. **(e)** HeLa cells were incubated with itaconate (4 mM) and the indicated MCT1/4 inhibitors (Su3118 10 μM, VB124 20 μM, AZD3965 1 μM). The uptake of itaconate were determined by LC-MS. **(f-k)** HEK 293T or U2OS cells were transfected with MCT1, MCT4 or their mutants. 24 h later, the endogenous or extracellular lactate level was measured at 6 h by LC-MS (f-i) or the Lactate-Glo™ Assay (Promega#J5021) (j, k). **(l)** Itaconate exists in both monocarboxylic and dicarboxylic forms at indicated pH, the percentage of monocarboxylic forms was listed according to the Henderson-Hasselbalch equation with pKa = 5.55, PubChem CID 811. **(m)** pH detection with dual-excitation ratiometric pH indicator BCECF-AM (5 μM) in BMDM with 100ng/ml LPS treatment for 22 h. Data are shown as the mean ± SD (d-k, m). Independent biological replicates-n=3 (d, f, g, j, k, m); n=4 (b, c, e, h, i). A two-tailed Student's t-test was conducted for pairwise comparisons (d, m), while one-way ANOVA was used for multiple comparisons involving a single independent variable (b-k).

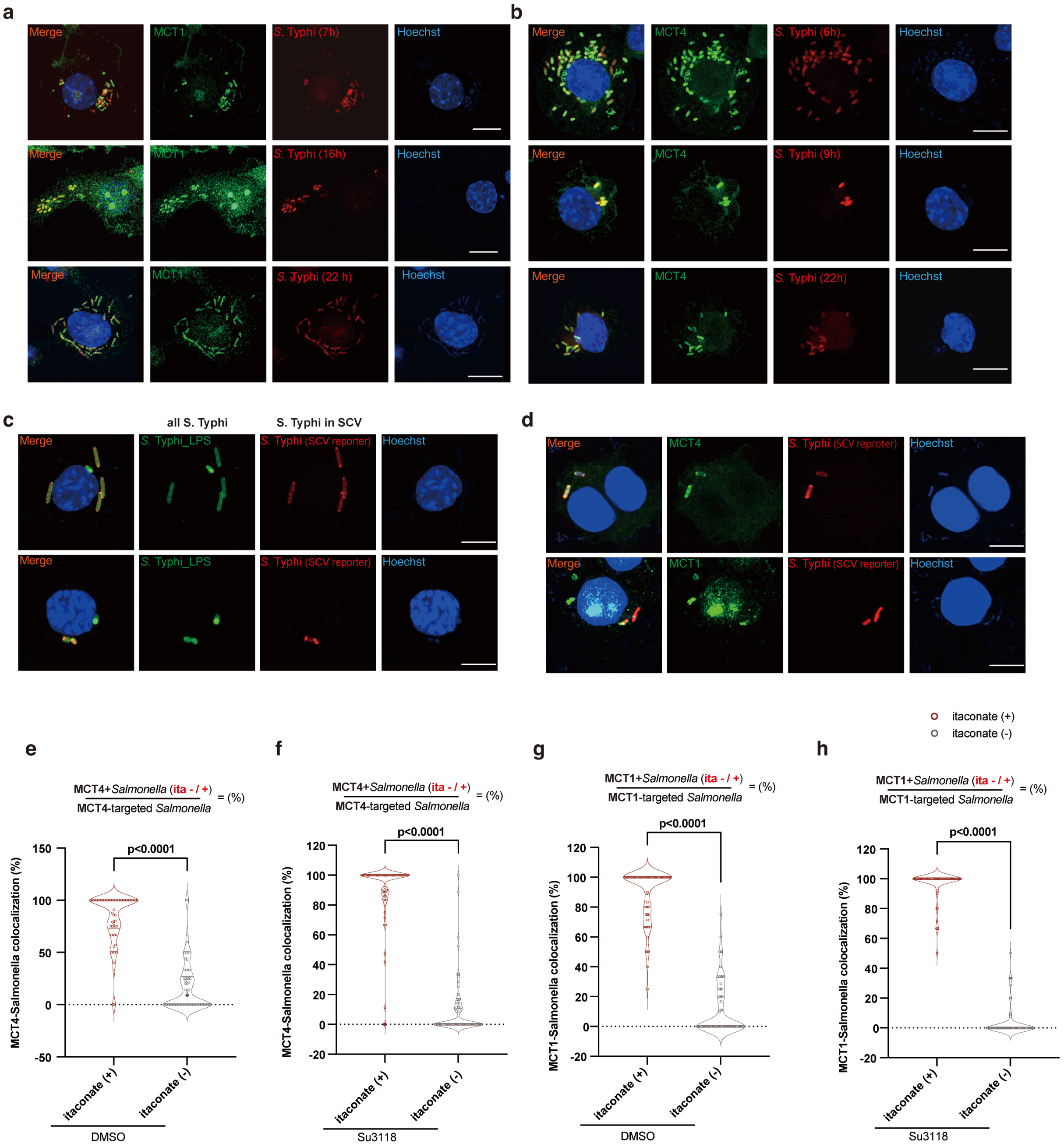

a
Merge
MCT1
S. Typhi (7h)
Hoechst
S. Typhi (16h)
S. Typhi (22 h)
b
MCT4
S. Typhi (6h)
S. Typhi (9h)
S. Typhi (22h)
c
all S. Typhi
S. Typhi in SCV
S. Typhi_LPS
S. Typhi (SCV reporter)
d
S. Typhi (SCV reproter)
itaconate (+)
itaconate (-)
e
MCT4+Salmonella (ita - / +) / MCT4-targeted Salmonella = (%)
MCT4-Salmonella colocalization (%)
p<0.0001
DMSO
f
Su3118
g
MCT1+Salmonella (ita - / +) / MCT1-targeted Salmonella = (%)
MCT1-Salmonella colocalization (%)
h

Supplementary Fig. 7 MCT1 and MCT4 transport itaconate into *Salmonella*-containing vacuole. (a-d) MCT1 and MCT4 are co-located with *Salmonella* containing vacuole. **(a, b)** RAW264.7 cells infected with *S*. Typhi expressing mScarlet (Red) for the indicated time points were fixed, incubated with Hoechst (Blue) to visualize nuclei and anti-MCT1/4 along with Alexa 488-conjugated anti-rabbit antibody (Green), and imaged under a confocal microscope. **(c)** The SCV reporter strain *S*. Typhi (SCV reporter) works in cells. RAW264.7 cells infected with a SCV reporter strain *S*. Typhi (SCV reporter) (Red) for 3 h were fixed, stained with Hoechst (Blue) to mark nuclei and LPS (Green) to mark all bacteria in the cytosol and SCV. **(d)** RAW264.7 cells infected with a SCV reporter strain *S*. Typhi (SCV) (Red) for 3 h were fixed, stained with Hoechst (blue) and MCT1/4 antibodies (Green). Scale bar, 10 μm. **(e-h)** Itaconate is prone to target MCT1/4-bound *Salmonella*. This is related to Figure 6e- 6h. RAW264.7 cells treated with DMSO/Su3118 and infected by *S*. Typhi encoding mScarlet (Red) and encoding itaconate eGFP biosensor (Green) for 16 h were fixed, stained with Hoechst (Blue) and incubated with MCT1/4 antibodies (Cyan), and imaged. More than 60 cells were collected unbiasedly. In each cell, we counted the total number of *Salmonella*, the number of MCT1/4-undecoraed/decorated *Salmonella* (MCT -/+) and the number of itaconate-untargeted/targeted-*Salmonella* (ita -/+). Then the ratio of MCT1/4-decorated *Salmonella* (ita -/+) was calculated: $\% = \frac{\mathrm{MCT}+\mathit{Salmonella}(\mathrm{ita}-/+)}{\mathrm{MCT}-\mathrm{targeted}\ \mathit{Salmonella}}$. Each dot represents the indicated ratio within one cell. Scale bar, 10 μm. Independent biological replicates, n=58 (e), n=64 (f), n=65 (g, h). A two-tailed Student's t-test was conducted for pairwise comparisons (e-h).

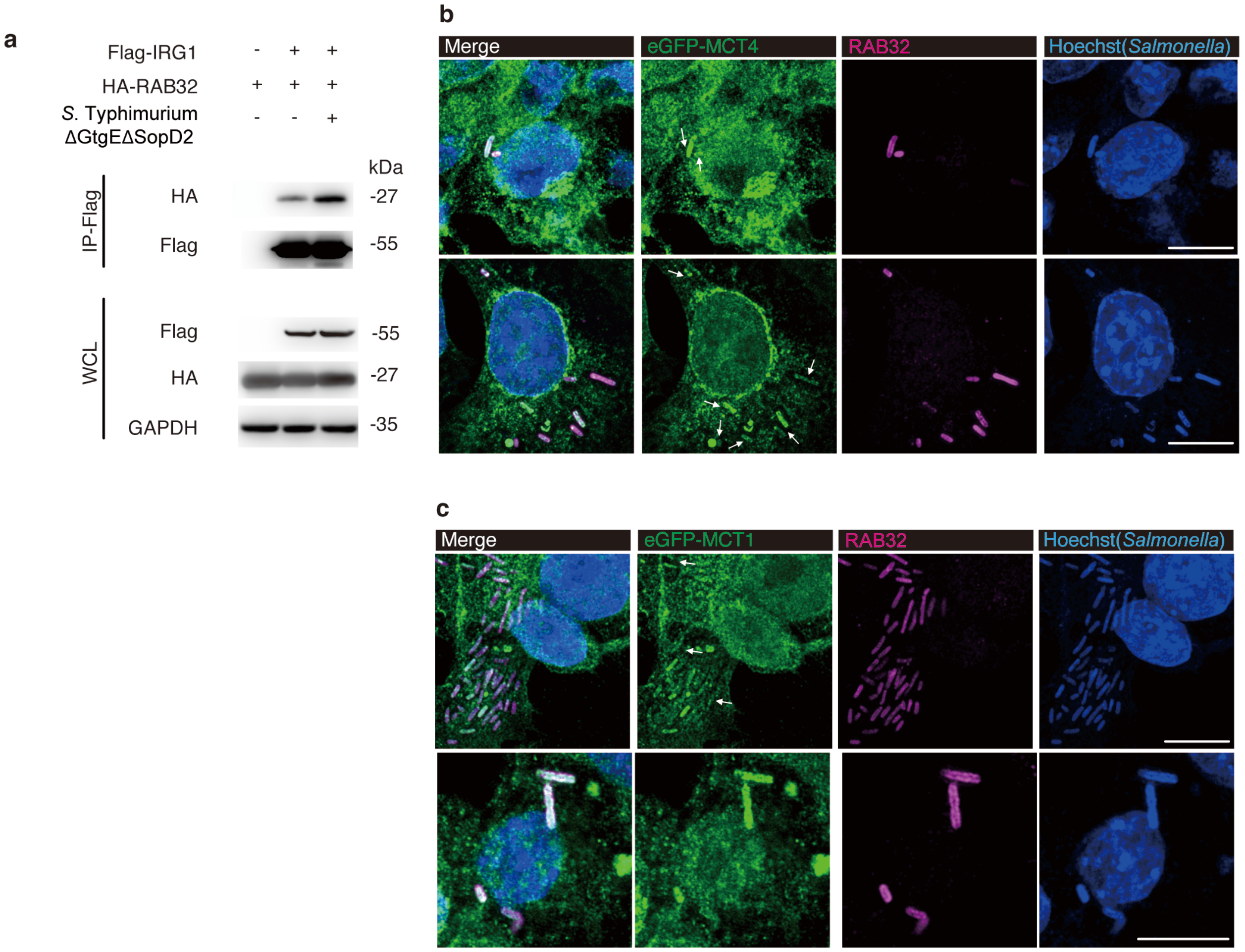

Supplementary Fig. 8 RAB32 and MCT1/4 are co-located at *Salmonella*-containing vacuole. (a) HEK 293T cells transfected with HA-RAB32, Flag-IRG1 (a). The whole cell lysates (WCL) were analyzed by immunoblotting with anti-HA, anti-Flag, or anti-GAPDH antibodies. The interaction of RAB32 and IRG1 (a) was examined by the co-immunoprecipitation assay. **(b, c)** eGFP- MCT1/4 (Green) stable RAW264.7 cell lines infected by *S*. Typhi for 22 h were fixed, stained with Hoechst (Blue) to mark *Salmonella* and nuclei and stained with RAB32 (Magenta) antibody along with Alexa 647-conjugated anti-rabbit antibody, and imaged. Scale bar, 10 μm.

Table S1. List of plasmids used in this study

NO.	Plasmids	Reference
pD1	eGFP-itaconate-biosensor	this study
pD3	nanoluciferase-itaconate-biosensor	this study
pD257	lenti-CMV-IRG1-Myc	this study
pD11	lenti-CMV-IRG1-eGFP	this study
pD29	mcmv-mMCT1-3×Flag	this study
pD3101	mcmv-mMCT4-3×Flag	this study
pD31	pCDNA3.1-hMCT1-Flag	this study
pD32	pECMV-hMCT4-3xFlag	this study
pD389	pBAD24-mscarlet SCV reporter	this study
pD8	pRK5-IRG1-Flag	this study
pD387	lenti-CMV-Flag-mMCT1	this study
pD388	lenti-CMV-Flag-mMCT4	this study
pD411	lenti-CMV-eGFP-mMCT1	this study
pD412	lenti-CMV-eGFP-mMCT4	this study
pD279	pcDNA3.1-HA-mMCT1	this study
pD362	pcDNA3.1-HA-mMCT4	this study
pD184	pcmv-HA-RAB32	this study
