# Supplementary material for: Itaconate transport across the plasma membrane and Salmonella-containing vacuole via MCT1/4 modulates macrophage antibacterial activity
Source: Nat Commun. 2025 Nov 26;16:10551. doi: 10.1038/s41467-025-65582-6 (PMC12658120; doi:10.1038/s41467-025-65582-6)
Supplement: Supplementary file 2 — Reporting Summary [file 41467_2025_65582_MOESM2_ESM.pdf]

nature portfolio

Corresponding author(s): Meixin Chen

Last updated by author(s): 9/12/2025

Reporting Summary

Nature Portfolio wishes to improve the reproducibility of the work that we publish. This form provides structure for consistency and transparency in reporting. For further information on Nature Portfolio policies, see our Editorial Policies and the Editorial Policy Checklist.

Statistics

For all statistical analyses, confirm that the following items are present in the figure legend, table legend, main text, or Methods section.

n/a	Confirmed	
☐	☒	The exact sample size (*n*) for each experimental group/condition, given as a discrete number and unit of measurement
☐	☒	A statement on whether measurements were taken from distinct samples or whether the same sample was measured repeatedly
☐	☒	The statistical test(s) used AND whether they are one- or two-sided<br>*Only common tests should be described solely by name; describe more complex techniques in the Methods section.*
☒	☐	A description of all covariates tested
☐	☒	A description of any assumptions or corrections, such as tests of normality and adjustment for multiple comparisons
☐	☒	A full description of the statistical parameters including central tendency (e.g. means) or other basic estimates (e.g. regression coefficient) AND variation (e.g. standard deviation) or associated estimates of uncertainty (e.g. confidence intervals)
☐	☒	For null hypothesis testing, the test statistic (e.g. *F*, *t*, *r*) with confidence intervals, effect sizes, degrees of freedom and *P* value noted<br>*Give P values as exact values whenever suitable.*
☒	☐	For Bayesian analysis, information on the choice of priors and Markov chain Monte Carlo settings
☒	☐	For hierarchical and complex designs, identification of the appropriate level for tests and full reporting of outcomes
☒	☐	Estimates of effect sizes (e.g. Cohen's *d*, Pearson's *r*), indicating how they were calculated

Our web collection on statistics for biologists contains articles on many of the points above.

Software and code

Policy information about availability of computer code

Data collection	Luciferase intensity of the itaconate biosensor was measured by<br>the Tecan Infinite®M1000 plate reader.<br>Western blotting were visualized using<br>Amersham ImageQuant 800 Western blot imaging systems.<br>Slides were imaged using<br>an inverted confocal laser scanning microscope Zeiss LSM 900.<br>Data of Real-time PCR was collected with<br>CFX96 Touch Real-Time PCR Detection System.<br>Data acquisition was conducted with a UHPLC system (Vanquish, Thermo Scientific) coupled to an Orbitrap mass spectrometer (Exploris 480, Thermo Scientific).<br>Liposomes were examined with a Tundra microscope operating at 100 kV,<br>with an electron dose of 40 electrons/Å for imaging. Images were captured using a CETA F camera.<br>Proteoliposomes' images were captured using optical diffraction tomography (ODT) with a live MH-HoliView Panoramic super-resolution microscope (Pellucid Optics Technology, Nantong Co., LTD).<br>The number of intracellular Salmonella per cell was quantified and analyzed with Opera Phenix Plus High Content Screening System (Shenzhen Bay Laboratory Imaging Platform).<br>FACS Data were acquired on a flow cytometer (Attune Nxt, Thermo).
Data analysis	Data acquisition and analysis of LC-MS were performed using<br>the SCIEX OS software (version 1.7.0, AB Sciex, Warrington, UK).<br>Data of the indicated cases were analyzed

by GraphPad Prism 9.0 software.
Images were analyzed by
Image J and ZEN Microscopy Software.
Metabolite annotation was performed using MetDNA (http://metdna.zhulab.cn/).

For manuscripts utilizing custom algorithms or software that are central to the research but not yet described in published literature, software must be made available to editors and reviewers. We strongly encourage code deposition in a community repository (e.g. GitHub). See the Nature Portfolio guidelines for submitting code & software for further information.

Data

Policy information about availability of data

All manuscripts must include a data availability statement. This statement should provide the following information, where applicable:

- Accession codes, unique identifiers, or web links for publicly available datasets
- A description of any restrictions on data availability
- For clinical datasets or third party data, please ensure that the statement adheres to our policy

Our data are available in the main text and supplementary materials.The Source Data file is also provided. This paper does not report any original codes.

Research involving human participants, their data, or biological material

Policy information about studies with human participants or human data. See also policy information about sex, gender (identity/presentation), and sexual orientation and race, ethnicity and racism.

Reporting on sex and gender	PBMCs were obtained from healthy male donors only. This is a limitation of the study.
Reporting on race, ethnicity, or other socially relevant groupings	This is a limitation of the study. These variables were not analyzed in this study.
Population characteristics	Human peripheral blood was obtained from healthy adult donors (ages 26, 28, male). All donors were free of known infectious or chronic diseases at the time of collection.
Recruitment	PBMCs were obtained from anonymized healthy adult donors via a commercial supplier (MILECELL BIO), which ensured informed consent and ethical compliance in accordance with applicable regulations.
Ethics oversight	PBMCs were obtained from a commercial provider (MILECELL BIO), which confirmed that informed consent was obtained and all procedures were approved by an independent ethics review board in accordance with applicable regulations.<br>Details:<br>hPB Leukopak report # PX24100304; COA-MY-S-2410016-02<br>hPB Leukopak report # PX25031805; COA-MY-S-2503207-01

Note that full information on the approval of the study protocol must also be provided in the manuscript.

Field-specific reporting

Please select the one below that is the best fit for your research. If you are not sure, read the appropriate sections before making your selection.

☒ Life sciences ☐ Behavioural & social sciences ☐ Ecological, evolutionary & environmental sciences

For a reference copy of the document with all sections, see nature.com/documents/nr-reporting-summary-flat.pdf

Life sciences study design

All studies must disclose on these points even when the disclosure is negative.

Sample size	Sample size was determined according to the previous experience (PMID: 32703879; PMID: 37640963).
Data exclusions	No data was excluded from this study.
Replication	All experiments were repeated at least 3 times with equivalent results.
Randomization	In all experiments, samples were randomly collected.
Blinding	For measurement of itaconate or other metabolites by LC-MS, the operation was blinded to the nature of the samples.<br>For other experiments, this was not the case as it was not possible for the operators to do so.

Reporting for specific materials, systems and methods

We require information from authors about some types of materials, experimental systems and methods used in many studies. Here, indicate whether each material, system or method listed is relevant to your study. If you are not sure if a list item applies to your research, read the appropriate section before selecting a response.

Materials & experimental systems

n/a	Involved in the study
☐	☒ Antibodies
☐	☒ Eukaryotic cell lines
☒	☐ Palaeontology and archaeology
☐	☒ Animals and other organisms
☒	☐ Clinical data
☒	☐ Dual use research of concern
☒	☐ Plants

Methods

n/a	Involved in the study
☒	☐ ChIP-seq
☒	☐ Flow cytometry
☒	☐ MRI-based neuroimaging

Antibodies

Antibodies used

Antibodies used were
anti-MCT1 (GXP457728, genxspan; Novus, NBP1-59656),
anti-MCT4 (22787-1-AP, proteintech),
anti-IRG1 (19857S, CST),
anti-NRF2 (12721S, CST),
anti-RAB32(GTX130477, Genetex)
anti-Flag M2 (F1804, Sigma),
anti-HA (26183, Thermo)
anti-b-actin (A1978, sigma),
anti-GAPDH (5174S, CST),
Goat anti-Mouse IgG (H+L) Secondary Antibody (C31430100, thermos,
Goat anti-Rabbit IgG (H+L) Secondary Antibody (C31460100, thermos).
488-conjugated Goat Anti-Mouse IgG(H+L) (SA00013-1),
488-conjugated Goat Anti-Rabbit IgG(H+L) (SA00013-2),
594-conjugated Goat Anti-Mouse IgG(H+L) (SA00013-3),
594-conjugated Goat Anti-Rabbit IgG(H+L) (SA00013-4) were from proteintech.

Validation

Whenever possible, antibodies were validated with positive and negative controls.

Eukaryotic cell lines

Policy information about cell lines and Sex and Gender in Research

Cell line source(s)

RAW264.7 (#SCSP-5036) and HeLa cells (#TCHu187) were obtained from
National Collection of Authenticated Cell Cultures.
HEK 293T cell line was kindly provided by
Professor Gong Cheng (Tsinghua University).
DC2.4 cells as kindly provided by
Professor Xun Sun (West China School of Pharmacy, Sichuan University).

Authentication

Cell lines were not authenticated.

Mycoplasma contamination

All cells lines were monthly tested for Mycoplasma and were always negative.

Commonly misidentified lines
(See ICLAC register)

Not commonly misidentified cell lines were used in this study.

Animals and other research organisms

Policy information about studies involving animals; ARRIVE guidelines recommended for reporting animal research, and Sex and Gender in Research

Laboratory animals

Irg1-/- mice (C57BL/6NJ-Acod1<em1(IMPC)J>/J; stock No: 029340)
were purchased from The Jackson Laboratory. Mct4-/+ mice (#S-KO-15319) were purchased from Cyagen. Mct4 homozygous mice were obtained by breeding Mct4 heterozygous mice (#S-KO-15319) and confirmed through genotyping.
8-to-12-week-old animals was used. Mice were sacrificed via CO_2 inhalation, following the protocols established by the Institutional Animal Care and Use Committee at Shenzhen Bay Laboratory. Mice experiments included both sexes, with age- and sex-matched pairs for each genotype in all conditions.

Wild animals	No wild animals were used.
Reporting on sex	Male and female mice were used and equal numbers were assigned to each category.
Field-collected samples	No field-collected samples were used.
Ethics oversight	The animal care and experimental protocols underwent thorough review and received the approval from the Institutional Animal Care and Use Committee at Shenzhen Bay Laboratory (SZBL).

Note that full information on the approval of the study protocol must also be provided in the manuscript.

Plants

Seed stocks	N/A
Novel plant genotypes	N/A
Authentication	N/A
